# Blind Compensation of *I*/*Q* Impairments in Wireless Transceivers

**DOI:** 10.3390/s17122948

**Published:** 2017-12-19

**Authors:** Mohsin Aziz, Fadhel M. Ghannouchi, Mohamed Helaoui

**Affiliations:** iRadio Lab, Department of Electrical and Computer Engineering, University of Calgary, 2500 University Dr. NW, Calgary, AB T2N 1N4, Canada; fghannou@ucalgary.ca (F.M.G.); mhelaoui@ucalgary.ca (M.H.)

**Keywords:** cumulative distribution function, demodulator, direct conversion transceivers, *I*/*Q* imbalance, modulator

## Abstract

The majority of techniques that deal with the mitigation of in-phase and quadrature-phase (*I*/*Q*) imbalance at the transmitter (pre-compensation) require long training sequences, reducing the throughput of the system. These techniques also require a feedback path, which adds more complexity and cost to the transmitter architecture. Blind estimation techniques are attractive for avoiding the use of long training sequences. In this paper, we propose a blind frequency-independent *I*/*Q* imbalance compensation method based on the maximum likelihood (ML) estimation of the imbalance parameters of a transceiver. A closed-form joint probability density function (PDF) for the imbalanced *I* and *Q* signals is derived and validated. ML estimation is then used to estimate the imbalance parameters using the derived joint PDF of the output *I* and *Q* signals. Various figures of merit have been used to evaluate the efficacy of the proposed approach using extensive computer simulations and measurements. Additionally, the bit error rate curves show the effectiveness of the proposed method in the presence of the wireless channel and Additive White Gaussian Noise. Real-world experimental results show an image rejection of greater than 30 dB as compared to the uncompensated system. This method has also been found to be robust in the presence of practical system impairments, such as time and phase delay mismatches.

## 1. Introduction

Modern communication networks suffer from various imperfections that significantly degrade their performance. Transmitters (Tx) and receivers (Rx) employ local oscillators to up- and down-convert the in-phase (*I*) and quadrature-phase (*Q*) signals to the carrier frequency and baseband, respectively. However, due to the non-idealities of the local oscillators and mixers, there is an imbalance in the gain and phase of the up/down-converted *I*/*Q* signals. *I*/*Q* imbalance results in mirror frequency imaging [1]. This problem is quite prevalent in direct-conversion radio architecture where the image falls within the band of interest after down-conversion.

The effects of *I*/*Q* imbalance can be mitigated using signal processing techniques, instead of adding hardware components to the transceiver topology or making changes in the transceiver topology. Various methods have been proposed to mitigate the effects of modulator and/or demodulator imperfections [1,2,3,4,5,6,7,8,9,10,11,12,13,14,15]. These include both frequency-independent (e.g., [4,5,6,7,10,11,15]) and frequency-dependent models (e.g., [8]).

The method proposed in [1] employs two solutions to mitigate the effects of *I*/*Q* mismatch in OFDM receivers. The first method is based on least squares, while the second is an adaptive least mean squares based method using pilot tones. However, the effect of transmitter imbalances has not been considered. Similarly, the methods suggested in [2,3] aim to mitigate the joint effect of receiver *I*/*Q* imbalance in the presence of phase noise and carrier frequency offset, respectively. The imbalance parameters are estimated by minimizing the mean squared error. A maximum likelihood based estimation for transceiver’s *I*/*Q* imbalance is presented in [4] in the presence of Additive White Gaussian Noise (AWGN) channel. Many of these methods, however, require long training sequences to estimate the model coefficients. These techniques also require the disruption of signal transmission as the imbalanced signals need to be time and phase aligned with the training/input signal for accurate modeling.

Blind methods are attractive in cases where long training sequences are not available or need to be avoided. Blind and semi-blind methods have been proposed in literature [5,6,7,8,9,11] with the aim of mitigating these imperfections. A gradient-based adaption algorithm based on image suppression ratio has been suggested in [7] for low-IF transmitters. The method proposed in [8] uses second-order statistics, i.e., the circularity property of the signals, to eliminate the effect of *I*/*Q* imbalance in quadrature radio receivers. Similarly, the method proposed in [10] provides a maximum likelihood estimation of receiver *I*/*Q* imbalance parameters. A higher order statistics based method for blind transmitter *I*/*Q* imbalance calibration has been proposed in [11]. The authors make use of a diode detector in the feedback path for detecting the instantaneous envelope of the transmitted signal, for its simplicity. As pointed out by the authors, demodulator or mixer based circuits can also be used in the feedback path. However, a limitation of these methods is that they either consider the effect of the modulator or the demodulator *I*/*Q* imbalance, i.e., if the effect of the modulator imbalance is considered, the demodulator is considered as ideal and vice versa. Similarly, the authors in [12] investigated the effects of Tx *I*/*Q* imbalance in direct-conversion transmitters while considering a super-heterodyne receiver. This, however, eliminates the direct-conversion architecture’s advantage of low complexity and cost efficiency. Hence, the methods that can compensate for both the Tx and Rx impairments without the need for a change in the architecture are attractive for practical systems.

Methods that consider this combined effect have been presented in [13,14]. However, both of these techniques, similar to [4], are data-aided techniques that, as previously mentioned, reduce the throughput of the system. Blind compensation methods for both the transmitter and the receiver *I*/*Q* imbalance compensation have been proposed in [15,16]. The former relies upon carrier frequency offset to decouple these imbalances followed by Cholesky decomposition for the estimation procedure. While the later proposes a two-step blind compensation technique using marginalized particle filter, which suffers from high computational complexity. In addition, none of these techniques provides an experimental validation of the proposed methodologies and their resulting impairments in realistic scenarios using measurement set-ups.

This paper proposes a novel post-compensation methodology for the transceiver (Tx-Rx) impairments that has the advantage of joint blind compensation for both the modulator and demodulator *I*/*Q* imbalances at the receiver, based on the statistics of the received signal. In addition, the proposed technique also eliminates the need for decoupling these imbalances. Statistics-based methods have been shown to perform reasonably well in the case of other front-end impairments such as power amplifier nonlinearity [17,18].

The proposed methodology requires only prior knowledge of the standard deviation of the actual *I* and *Q* signals and does not require pilots or training sequences. Another important feature of the proposed methodology is that it does not need a feedback path at the transmitter, resulting in a much simpler architecture. The restrictions for low cost and implementation size of the uplink scenario (mobile to base station communication) demand effective post-compensation schemes due to the difficulty in implementing complex compensation schemes in the mobile set, while maintaining computational and cost efficiency. Post compensation allows the base station receiver to apply the mitigation algorithm, as it has more computational resources and hardware flexibility than that of the mobile receiver system. The implementation of these methods helps avoid the feedback path at the transmitter saving valuable hardware resources such as demodulators, filters, and analog-to-digital converters (ADCs) and the resulting impairments.

The rest of the paper is organized as follows: Section 2 starts with a detailed model description and mathematical formulation, followed by the proposed mitigation strategy. Section 3 describes the performance evaluation of the proposed methodology using computer simulations. Section 4 provides a discussion regarding the proposed work. Section 5 presents the measurement setup and results obtained using the proposed methodology. Conclusions are summarized in Section 6.

## 2. Model Formulation and Mitigation Methodology

### 2.1. I/Q Impairment Model Formulation

**Figure 1:**
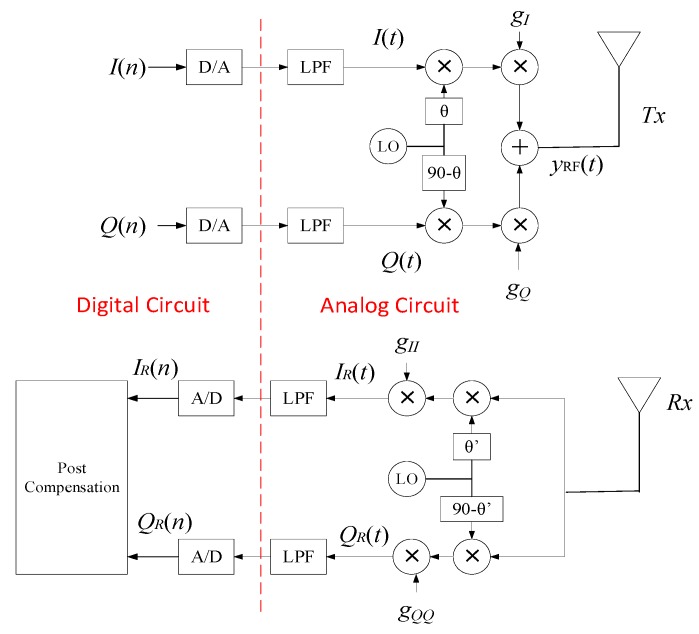
Block diagram of transceiver system with modulator’s and demodulator’s imperfections.

Figure 1 shows a simplified block diagram of a transceiver system with modulator and demodulator imperfections, along with the post-compensation block. In a typical transmitter, the baseband *I* and *Q* signals pass through the modulator and are up-converted to the desired radio frequency (RF). Ideally, the local oscillator should produce a 90 degrees phase difference between the *I* and the *Q* branches, while maintaining equal gain between the two branches. However, this difference is not exactly 90 degrees, due to the non-idealities of various components used for up- and down-conversion, resulting in phase imbalance. Similarly, a gain mismatch is introduced between the *I* and the *Q* branches of the modulator/-demodulator, which also affects the quality of the signal. The combined effect of the gain and phase imbalances is referred to as *I*/*Q* imbalance. The RF output of the modulator can be represented as: $y_{RF}\left(t\right)=Re\left\{\left(I_{T}\left(t\right)+jQ_{T}\left(t\right)\right)e^{2\pi ft}\right\}$, where *f* is the carrier frequency and *t* is the time. The baseband equivalent of the output *I* and *Q* of the modulator can be expressed as [11]:

(1) $$I_{T}(t)=g_{I}\cos(\theta )I(t)+g_{Q}\sin(\theta )Q(t)$$

(2) $$Q_{T}(t)=g_{I}\sin(\theta )I(t)+g_{Q}\cos(\theta )Q(t)$$

where $g_{I}$ and $g_{Q}$ are the transmitter’s gain imbalance parameters in the *I* and the *Q* paths, respectively. The phase imbalance i.e., *φ* is split equally between the two branches i.e., *θ* = *φ*/2. The transmitted signal after passing through an AWGN channel, in matrix form, can be written as:

(3) $$y_{T}=\varsigma Tx+n$$

where

(4) $$\varsigma =\sqrt{\frac{g_{I}^{2}+g_{Q}^{2}}{2}};\;T=\sqrt{\frac{2}{\left(1+g^{2}\right)}}\left(\begin{array}{cc} g\cos(\theta ) & \sin(\theta )\\ g\sin(\theta ) & \cos(\theta ) \end{array}\right)$$

Here $g=g_{I}/g_{Q}$, $y_{T}={\left[I_{T}\left(t\right)\;Q_{T}\left(t\right)\right]}^{T}$ is the imbalanced signal at the output of the modulator, while $x={\left[I\left(t\right)\;Q\left(t\right)\right]}^{T}$ is the input signal to the modulator. **T** is the matrix of modulator’s imbalance parameters, $n={\left[n_{I}\left(t\right)\;n_{Q}\left(t\right)\right]}^{T}$ is the noise vector, and $n_{I}\left(t\right)$ and $n_{Q}\left(t\right)$ are the noise components in the *I* and the *Q* branches respectively. This signal is then demodulated at the receiver, which also introduces an *I*/*Q* imbalance resulting in the following expressions for the received signals:

(5) $$I_{R}(t)=g_{II}\cos(\theta^{\prime })I_{T}(t)+g_{QQ}\sin(\theta^{\prime })Q_{T}(t)+{\tilde{n}}_{I}(t)$$

(6) $$Q_{R}(t)=g_{II}\sin(\theta^{\prime })I_{T}(t)+g_{QQ}\cos(\theta^{\prime })Q_{T}(t)+{\tilde{n}}_{Q}(t)$$

where $g_{II}$ and $g_{QQ}$ are the receiver’s gain imbalance parameters in the *I* and *Q* components, respectively. Similar to the modulator, *θ*′ is the phase imbalance in each branch of the receiver. Defining $g^{\prime }=g_{II}/g_{QQ}$, the output of the demodulator can be written as:

(7) $$y_{R}=\varsigma^{\prime }Ry_{T}=\varsigma \varsigma^{\prime }RTx+\tilde{n}$$

where $y_{R}={\left[I_{R}\left(t\right)\;Q_{R}\left(t\right)\right]}^{T}$ is the signal at the output of the demodulator, **R** is the matrix of the receiver’s imbalance parameters and

(8) $$\varsigma^{\prime }=\sqrt{\frac{g_{II}^{2}+g_{QQ}^{2}}{2}};\;R=\sqrt{\frac{2}{\left(1+{g^{\prime }}^{2}\right)}}\left(\begin{array}{cc} g^{\prime }\cos(\theta^{\prime }) & \sin(\theta^{\prime })\\ g^{\prime }\sin(\theta^{\prime }) & \cos(\theta^{\prime }) \end{array}\right)$$

Since, the *I*/*Q* imbalance considered in this case is linear with respect to the modeling parameters, the combined baseband model of the *I* and *Q* components under the effect of both the imbalances can be written as:

(9) $$y_{R}=\varsigma \varsigma^{\prime }\sqrt{\frac{2}{\left(1+g^{2}\right)}}\sqrt{\frac{2}{\left(1+{g^{\prime }}^{2}\right)}}\left(\begin{array}{cc} g^{\prime }\cos(\theta^{\prime }) & \sin(\theta^{\prime })\\ g^{\prime }\sin(\theta^{\prime }) & \cos(\theta^{\prime }) \end{array}\right)\left(\begin{array}{cc} g\cos(\theta ) & \sin(\theta )\\ g\sin(\theta ) & \cos(\theta ) \end{array}\right)x+\tilde{n}$$

Or simply

(10) $$y_{R}=Ax+\tilde{n}=y+\tilde{n}$$

where $y={\left[I_{y}\left(t\right)\;Q_{y}\left(t\right)\right]}^{T}$ and **A** is the matrix containing the joint imbalance parameters. Matrix **A** is defined as:

(11) $$A=\left(\begin{array}{cc} \alpha_{1} & \beta_{1}\\ \alpha_{2} & \beta_{2} \end{array}\right)$$

where

(12) $$\alpha_{1}=\varsigma \varsigma^{\prime }\sqrt{\frac{2}{\left(1+g^{2}\right)}}\sqrt{\frac{2}{\left(1+{g^{\prime }}^{2}\right)}}\left(gg^{\prime }\cos(\theta )\cos(\theta^{\prime })+g\sin(\theta )\sin(\theta^{\prime })\right)$$

(13) $$\beta_{1}=\varsigma \varsigma^{\prime }\sqrt{\frac{2}{\left(1+g^{2}\right)}}\sqrt{\frac{2}{\left(1+{g^{\prime }}^{2}\right)}}\left(g^{\prime }\cos(\theta^{\prime })\sin(\theta )+\sin(\theta^{\prime })\cos(\theta )\right)$$

(14) $$\alpha_{2}=\varsigma \varsigma^{\prime }\sqrt{\frac{2}{\left(1+g^{2}\right)}}\sqrt{\frac{2}{\left(1+{g^{\prime }}^{2}\right)}}\left(gg^{\prime }\cos(\theta )\sin(\theta^{\prime })+g\sin(\theta )\cos(\theta^{\prime })\right)$$

(15) $$\beta_{2}=\varsigma \varsigma^{\prime }\sqrt{\frac{2}{\left(1+g^{2}\right)}}\sqrt{\frac{2}{\left(1+{g^{\prime }}^{2}\right)}}\left(g^{\prime }\sin(\theta^{\prime })\sin(\theta )+\cos(\theta^{\prime })\cos(\theta )\right)$$

Using this imbalance model, the next step is to derive a closed-form probability density function (PDF) expression of the output signals in terms of the imbalanced parameters and find an estimate of matrix **A** using the maximum likelihood (ML) estimation.

### 2.2. Closed-form PDF of the Imbalanced Signal

The following assumptions were made before deriving the PDF of the demodulated signal:

*A*1: The *I* and *Q* components are jointly Gaussian and independent, i.e., (*E*[*IQ*] = *E*[*I*]E[*Q*]), where *E*[.] is the expectation operation.

*A*2: The input *I* and *Q* signals have similar statistics, i.e., both components have zero means (${µ}_{I}$ = ${µ}_{Q}=0$) and same variances ($\sigma_{I}^{2}=\sigma_{Q}^{2}=\sigma^{2}$).

These assumptions result in the covariance matrix of **x** to be diagonal i.e., $C_{x}=\sigma^{2}I$, where **I** represents identity matrix and are valid for practical communication signals without making significant changes in system specifications. The assumption of *I* and *Q* signals being independent is considered in many works (e.g., reference [8] in this work and references [20–25] found in [11]). Orthogonal frequency division multiplexing (OFDM) signals have been shown to exhibit Gaussian characteristics [19]. The authors in [11] also assume similar statistics of *I* and *Q* signals.

For *N* samples of the received signal, the *N*-point PDF of **y** can be written as [10]:

(16) $$f_{y}(y;\Gamma )=\prod_{i=1}^{N}\frac{1}{2\pi \sqrt{\;|C_{y}(\Gamma )|}}\exp(-\frac{1}{2}y_{i}^{T}C_{y}^{-1}(\Gamma )y_{i})$$

where $C_{y}$ denotes the covariance matrix of **y** and $Г={\left[\alpha_{1}\;\beta_{1}\;\alpha_{2}\;\beta_{2}\right]}^{T}\;$ is the vector of unknown imbalance parameters defined in Equations (12)–(15). Using the expressions for the covariance matrix of **y** i.e., $C_{y}$ and its determinant, the joint PDF of $I_{y}$ and $Q_{y}$ takes the following form (see Appendix A for derivation):

(17) $$f_{y}(y;\Gamma )=\frac{1}{{\left(2\pi \sigma^{2}|\gamma |\right)}^{N}}e^{-\left(\frac{1}{2\sigma^{2}\gamma^{2}}\sum_{i=1}^{N}p(i)\right)}$$

where *σ* represents the standard deviation of the *I* and *Q* signals, |*γ*| is the determinant of **A**. An auxiliary set of variables has been used for mathematical simplicity, which are defined as:

(18) $$p(i)=p_{1}^{2}(i)+p_{2}^{2}(i)$$

(19) $$p_{1}(i)=\beta_{2}I_{y}(i)-\beta_{1}Q_{y}(i)$$

(20) $$p_{2}(i)=-\alpha_{2}I_{y}(i)+\alpha_{1}Q_{y}(i)$$

The analytical closed-form expression for the PDF of the imbalanced signal obtained using Equation (17) is herein referred to as the ‘derived’ PDF. It should be noted here that the noise has not been included in the analysis i.e., $y_{R}$ = **y** in Equation (10). A maximum likelihood estimation of the imbalance parameters is obtained using this PDF. In the presence of noise, however, the PDF of the imbalanced signal $y_{R}$ is given below, where * represents convolution of the two PDFs [20]:

(21) $$f_{y_{R}}(y_{R};\Gamma )=f_{y}(y;\Gamma )*f_{\tilde{n}}(\tilde{n};\Gamma )$$

### 2.3. Accuracy of Derived PDF

The accuracy of the derived PDF can be measured by various figures of merit, including the Kullback-Leibler (KL) divergence, which is the measure of similarity or dissimilarity between two PDFs. If we consider two discrete PDFs, $f\left(x_{k}\right)$ and $g\left(x_{k}\right)$, using *K* independent and identically distributed samples ${\left\{x_{k}\right\}}_{k=1}^{K}$, KL divergence can then be defined as [21]:

(22) $$D_{\mathrm{KL}}(f||g)=\sum_{k=1}^{K}f(x_{k})log\left(\frac{f(x_{k})}{g(x_{k})}\right)$$

A higher value of KL divergence indicates dissimilarity between the compared PDFs, while, a smaller value indicates similarity or closeness of the compared PDFs. Another figure of merit for comparing two PDFs is the Hellinger distance. The square of the Hellinger distance is defined as [22]:

(23) $$D_{H}^{2}(f||g)=\frac{1}{2}\sum_{k=1}^{K}{\left(\sqrt{f(x_{k})}-\sqrt{g(x_{k})}\right)}^{2}$$

The first step is to analyze the effect of the *I*/*Q* imbalances on the PDF of the signal. Kernel Density Estimation (KDE) is a well-established method for PDF estimation and hence has been used here for comparison. Table 1 compares the input signal’s PDF ($f_{\mathrm{in},\mathrm{KDE}}$) and the imbalanced signal’s PDF ($g_{\mathrm{KDE}}$) using the nonparametric KDE method for the metrics provided in Equations (22) and (23) for the noiseless case. These values of the KL divergence and Hellinger distance show the divergence in the PDFs of the modulator’s input signal and the demodulator’s output signal. This is due to the presence of the *I*/*Q* imbalance in the system, which changes the PDF of the input signal.

The next step is to determine the accuracy of the derived analytical expression in Equation (17). For this purpose, the derived PDF of the imbalanced signal $(f_{y}\left(y;Г\right)$ was compared to the KDE-based estimate of the output i.e., $g_{\mathrm{KDE}}$. In this case, Table 1 shows that the values of both the KL divergence and the Hellinger square distance are quite small for both Wideband Code Division Multiple Access (WCDMA) and Long-Term Evolution (LTE) signals, resulting in the conclusion that the derived PDF is very similar to the desired PDF.

**Figure 2:**
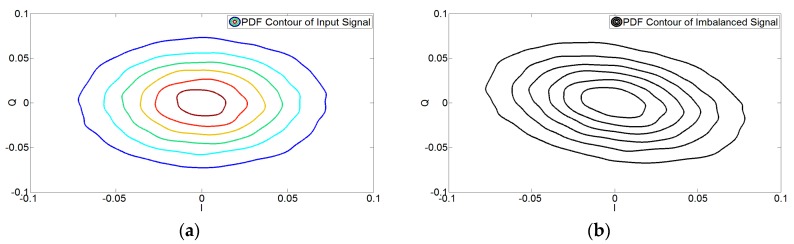
Contour plot for the PDF of Wideband Code Division Multiple Access (WCDMA) 1111 (**a**) input and (**b**) imbalanced output *I*/*Q* signals using the Kernel Density Estimation (KDE) method. A deviation from the input signal’s PDF can be seen due to *I*/*Q* imbalance.

**Figure 3:**
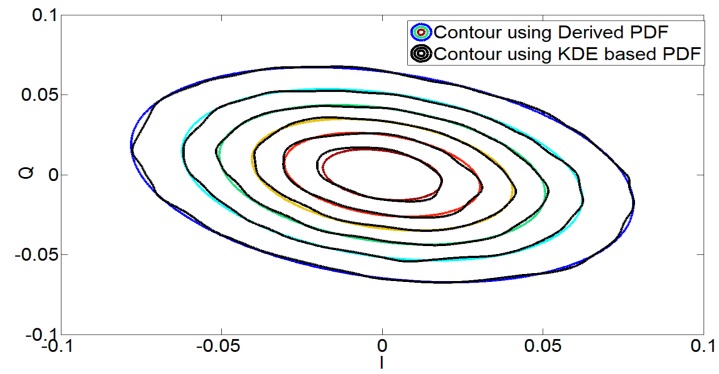
Contour plot for the PDF of imbalanced (*g* = 0.92 dB, *g*′ = 0.5 dB and *θ* = −6 degrees, *θ*′ = −2 degrees) signal using the KDE based method and the derived expression (17) for noiseless case. The derived PDF follows the PDF of the imbalanced signal obtained using the KDE method.

This is also evident from Figure 2 and Figure 3, which show the effect of *I*/*Q* imbalance on the PDF contours and the accuracy of the derived PDF of the output signal of the demodulator, respectively. It can be concluded from Table 1 and Figure 3 that there is an excellent correspondence between the derived and KDE-based PDFs of the imbalanced signals. It should be noted that the analysis provided here is for the PDF obtained in Equation (17) for the noiseless case i.e., derived PDF.

**Figure 4:**
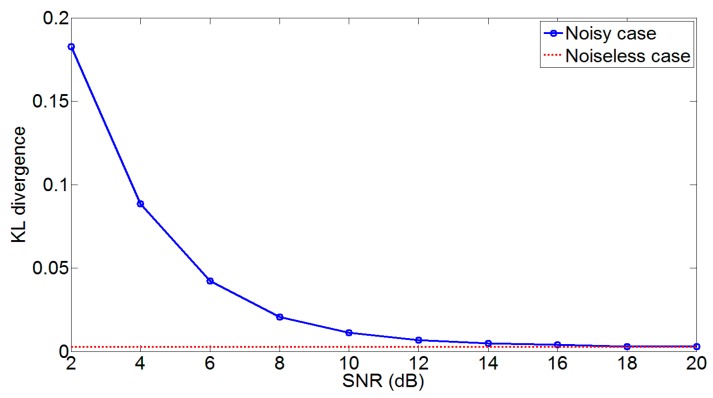
Kullback-Leibler (KL) divergence between KDE based estimate of $y_{R}$ and derived PDF for WCDMA 1111 signal. The dotted line shows the KL divergence for the noiseless case while the solid line includes the effect of noise.

Figure 4 shows the comparative analysis for the noisy case. It can be seen that the KL divergence between the derived PDF and KDE based estimate of $y_{R}$ is higher at lower Signal to Noise Ratios (SNRs). The reason being that the derived PDF does not consider the effects of noise and this results in a significant discrepancy between the two density functions. However, at around 12 dB SNR, the discrepancy reduces significantly and the KL divergence curve is close to the noiseless case (dotted line). Hence, the estimation of the imbalance signal is carried out using the derived PDF of $y_{R}$ without including the effects of noise as this leads to simplicity in the estimation procedure.

### 2.4. Parameter Estimation

Once the PDF of the imbalanced signals is obtained and verified, the next step is the estimation of imbalance parameters. These modeling parameters are obtained by maximizing the likelihood function or the derived PDF. Hence, the imbalance parameters are obtained by solving the system of following equations (see Appendix B for derivation):

(24) $$\frac{\beta_{2}}{2}(1^{T}.p)-\gamma (p_{2}{}^{T}.Q_{y})=0$$

(25) $$\frac{\alpha_{2}}{2}(1^{T}.p)-\gamma (p_{1}{}^{T}.Q_{y})=0$$

(26) $$\frac{\alpha_{1}}{2}(1^{T}.p)-\gamma (p_{1}{}^{T}.I_{y})=0$$

(27) $$\frac{\beta_{1}}{2}(1^{T}.p)-\gamma (p_{2}{}^{T}.I_{y})=0$$

where **1** is the unit vector containing all ones i.e., **1**_N×1_ = [1 1 …. 1]^T^; **p**, **p**_1_ and **p**_2_ are the vector representations of *p*(*i*), *p*_1_(*i*) and *p*_2_(*i*) from Equations (18)–(20), respectively for *i* = 1,…,*N*; and **I_y_** and **Q_y_** are the vector representations of $I_{y}$(*i*) and $Q_{y}\left(i\right)$.

By solving this set of four equations, the unknown imbalance parameters, i.e., $\alpha_{1}\;\beta_{1}\;\alpha_{2}$ and $\beta_{2}$ can be estimated. After an estimate is obtained, the estimated *I* and *Q* signals can finally be obtained with the following expression:

(28) $$\left(\begin{array}{c} \overset{\wedge }{I(i)}\\ \overset{\wedge }{Q(i)} \end{array}\right)={\left(\begin{array}{cc} {\overset{\wedge }{\alpha }}_{1} & {\overset{\wedge }{\beta }}_{1}\\ {\overset{\wedge }{\alpha }}_{2} & {\overset{\wedge }{\beta }}_{2} \end{array}\right)}^{-1}\left(\begin{array}{c} I_{R}(i)\\ Q_{R}(i) \end{array}\right)$$

It should be noted that we are able to estimate the final matrix **A** and not the transmitter and receiver imbalance matrices **T** and **R** individually. The Cramer-Rao Lower Bound (CRLB) for the proposed estimator is provided in Appendix C.

Solution of nonlinear equations: Here we discuss possible solutions to Equations (24)–(27) for the estimation of the imbalance parameters. Nonlinear multivariable optimization techniques, such as the simulated annealing algorithm [23] or the nonlinear system solver-based Levenberg-Marquardt method [24], can be used to solve these nonlinear system of equations. The simulated annealing algorithm relies on minimizing the system’s energy by lowering the temperature of the system until the convergence criterion is met. This technique is quite effective for non-convex systems, as it has the ability to avoid being stuck in the local minima. The complexity of the proposed methodology relies on the optimization technique required to estimate the imbalance parameters using Equations (24)–(27). Simulated Annealing algorithm has been used in this work to estimate the unknown parameters. A detailed analysis on the complexity of the simulated annealing algorithm has been provided in [25].

## 3. Simulation Results

### 3.1. Normalized Mean Squared Error and Image Suppression Evaluation

The figures of merit to assess the accuracy of the proposed method include the normalized mean squared error (NMSE), image suppression (IMG_sup_) and the bit error rate (BER). NMSE is an effective figure of merit for in-band performance analysis and is given by [26]:

(29) $$\mathrm{NMSE}(\mathrm{dB})=10\log_{10}(\frac{\sum_{l=1}^{L}|y_{act}(l)-y_{est}(l){|}^{2}}{\sum_{l=1}^{L}|y_{act}(l){|}^{2}})$$

where *y*_act_ is the desired/measured output, *y*_est_ is the output estimated by the proposed methodology and *L* is the length of data used for evaluation. Image suppression [7] (reciprocal of image rejection ratio) is the ratio of the image power to the desired signal power and measures the effectiveness of the algorithm to mitigate the *I*/*Q* imbalance.

(30) $${\mathrm{IMG}}_{\sup}(f)=10\log_{10}\left(\frac{P_{\mathrm{image}}(f)}{P_{\mathrm{signal}}(f)}\right)$$

where *P*_image_ is the power in the image band and *P*_signal_ is the power in the signal band and *f* denotes the frequency.

**Figure 5:**
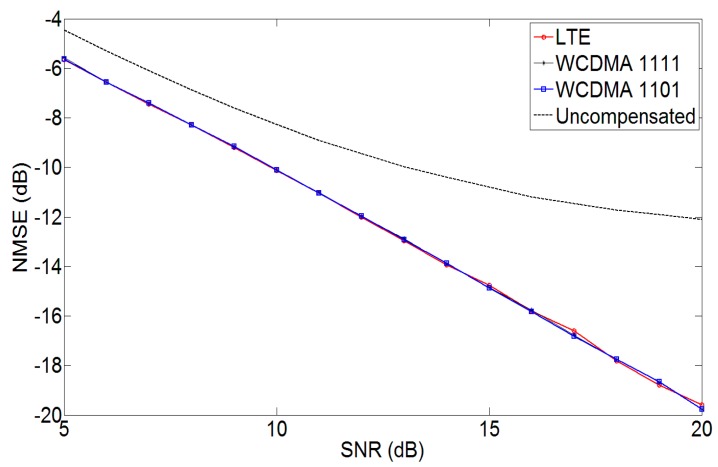
Normalized mean squared error (NMSE) vs. SNR using the proposed mitigation approach for *g* = *g*′ = 0.92 dB and *θ* = *θ*′ = −6 degrees.

Figure 5 shows the NMSE performance of the proposed methodology for various signals. The signals used for the evaluation of the proposed methodology were a 20 MHz four-carrier Wideband Code Division Multiple Access (WCDMA 1111) signal, a 9 MHz Long Term Evolution (LTE) signal and 20 MHz WCDMA 1101 signal. It can be seen in the figure, that with the proposed mitigation approach, the NMSE is considerably reduced as compared to the uncompensated case.

**Figure 6:**
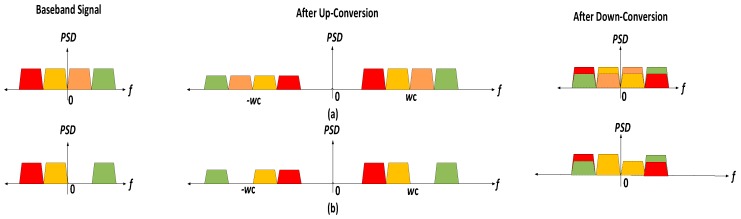
Mirror frequency Imaging (MFI) due to *I*/*Q* imbalance. (**a**) MFI for symmetric 1111 signal for which the power in the image band cannot be seen using PSD; (**b**) MFI for asymmetric signals and the power in the image band is visible.

As mentioned in [27], *I*/*Q* imbalance results in mirror frequency imaging, i.e., if the baseband/low-IF signal is up-converted to RF at a carrier frequency of *w*_c_, a mirror image of this signal is created at −*w*_c_ which when down-converted to baseband/low-IF results in cross talk between the signals, distorting the desired signal. For signals symmetric around the DC such as the WCDMA 1111 signal, the image lies exactly on top of the desired signal when down-converted. Hence, it is difficult to observe the image caused by the *I*/*Q* imbalance using the power spectral density. This can be seen in Figure 6a. However, as shown in Figure 6b, for signals not symmetric about DC, it is possible to observe the power in the image band using the spectrum of the signal.

**Figure 7:**
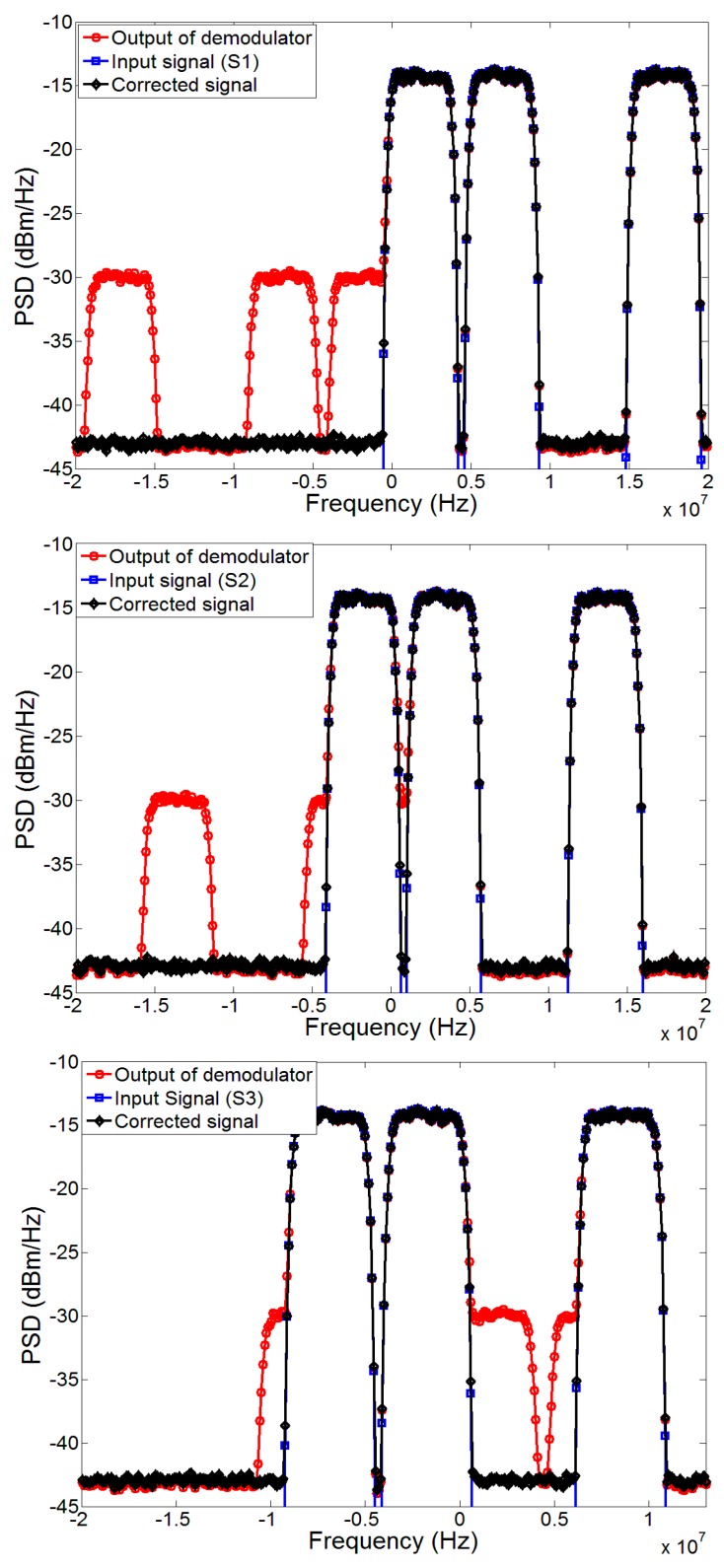
Power spectral density (PSD) of WCDMA 1101 (S1, S2 and S3) signals before and after correction, using the proposed method for *g* = 0.92 dB, *g*′ = 0.5 dB and *θ* = −6 degrees, *θ*′ = −2 degrees under 20 dB SNR.

For this reason, three cases of the WCDMA 1101 signals (namely S1, S2 and S3 as shown in Table 2) with different center frequencies (*f*_c_) are used to measure the image suppression performance of the proposed technique as shown in Figure 7. These signals are centered on different frequencies, i.e., *f*_c_ = 10 MHz, 5 MHz and around DC, respectively, and are used to evaluate the performance of the proposed methodology by observing the suppression in the image band. For WCDMA 1101 signal S1, the image lies out of band when down-converted, however, for WCDMA 1101 signals S2 and S3, the image band falls into the band of interest, degrading the signal quality.

The spectrum of compensated signal achieved by the proposed algorithm for these three WCDMA 1101 signals can also be seen in Figure 7 for *g* = 0.92 dB, *g*′ = 0.5 dB and *θ* = −6 degrees, *θ*′ = −2 degrees. It can be seen that by using the proposed methodology the image has been suppressed down to the noise floor leading to a reasonable image suppression as presented in Table 2.

Complexity: As mentioned earlier, the complexity of the proposed methodology depends on the optimization technique required to estimate the imbalance parameters. In the proposed work, we use simulated annealing algorithm as a case study to evaluate the speed and complexity of the proposed mitigation strategy. The number of data samples and the number of iterations required for convergence will be used as metrics to evaluate the speed of the algorithm. For a WCDMA 1111 signal, the proposed methodology requires as low as 3000 samples and 2820 iterations to converge to an NMSE of −19.37 dB under 20 dB SNR. Similarly, for a 9 MHz LTE signal, with 3000 samples, the algorithm converges to an NMSE of −19.43 dB in 2370 iterations.

### 3.2. BER Performance in the Presence of Multi-Path Channel

#### 3.2.1. Parameter Estimation under Known Channel

**Figure 8:**
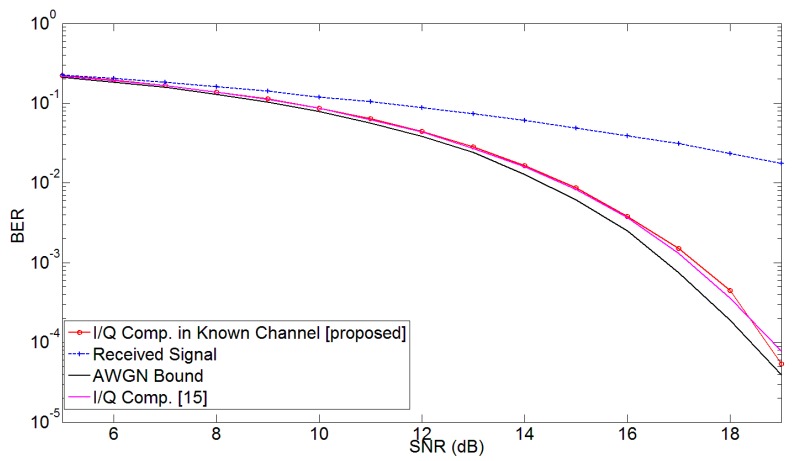
Bit error rate (BER) vs. SNR for the proposed model for 16 QAM orthogonal frequency division multiplexing (OFDM) signal with 1024 subcarriers for *g* = *g*′ = 0.92 dB and *θ* = *θ*′ = −6 degrees.

Another important factor in the evaluation of the proposed methodology is the effect of the wireless channel on the system performance. The problem of connection between transceiver impairments and channel estimation has been explained in [15,28,29]. In order to understand the effects of channel on the proposed mitigation strategy, an OFDM signal was generated to evaluate the performance of the proposed technique under the influence of wireless channel. Random data bits were generated and modulated using 16-Quadrature Amplitude Modulation (QAM). A 1024-point inverse Fourier transform was applied to this modulated signal. Modulator imbalance was applied to the signal and the signal was passed through a multi-path channel with the channel taps [0.866 + 0.5*j* 0.0643 + 0.0766*j* 0.0098 − 0.0017*j*] [15]. At the receiver, the demodulator imbalance parameters were applied; and, the signal was then fed to the post-compensation algorithm. The performance of the proposed mitigation methodology was tested after channel equalization, assuming that the channel state was known. The corrected signal was demodulated after application of Fast Fourier Transform (FFT); and the BER was computed. The BER performance vs. SNR is shown in Figure 8. It can be seen that the proposed method can reasonably estimate and correct for the joint modulator and demodulator impairments in the presence of multi-path channel and AWGN noise. The achieved BER using the proposed methodology is very close to the AWGN bound for all values of SNR.

#### 3.2.2. Parameter Estimation under Unknown Channel

In the previous section, the imbalance parameters were estimated after removing the channel effect assuming that the channel response was known. The next step, is to understand the effect of channel on the PDF of the output signal and estimate the *I*/*Q* imbalance parameters in the presence of unknown wireless channel response. In order to understand this, the following definitions are made:

$g_{\mathrm{in}}$: KDE estimate of the input signal’s PDF.

$g_{\mathrm{CH}}$: KDE estimate of the output signal’s PDF under the influence of Tx and Rx impairments and multi-path channel.

$g_{\mathrm{IQ}}$: KDE estimate of the output signal’s PDF under the influence of Tx and Rx impairments alone in AWGN channel.

$g_{S}$: KDE estimate of the output signal’s PDF under the influence of Tx and Rx impairments and multipath channel effects after gain and phase synchronization.

**Figure 9:**
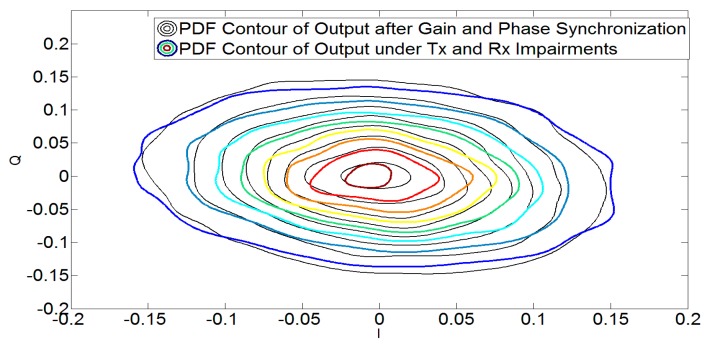
Contour plot for the PDF of 16-QAM OFDM *I*/*Q* signals using the KDE method under multipath channel and 20 dB SNR. The colored contours show the variations in PDF due to Tx and Rx *I*/*Q* imbalance only under Additive White Gaussian Noise (AWGN). The black contours show the PDF of the output signal under the influence of *I*/*Q* imbalance and channel after gain and phase synchronization.

**Figure 10:**
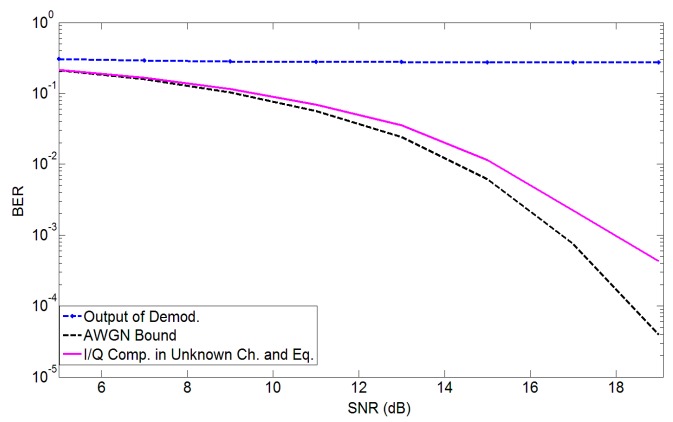
BER vs. SNR for the proposed model for 16 QAM OFDM signal with 1024 subcarriers for *g* = *g*′ = 0.5 dB and *θ* = *θ*′ = −2 degrees.

Table 3 compares these PDFs to understand the effects of wireless channel on signal’s PDF. KL divergence and square of Hellinger distance are higher when comparing $g_{\mathrm{in}}$ with $g_{\mathrm{CH}}$. This is due to the combined effect of transceiver impairments and channel. These merits are comparatively lower when comparing $g_{\mathrm{in}}$ with $g_{\mathrm{IQ}}$ as $g_{\mathrm{IQ}}$ only considers the effects of transceiver impairments alone and not the multi-path channel. However, if we perform gain and phase synchronization on the output signal under the influence of transceiver’s impairments and multipath channel, we obtain $g_{S}$. Table 3 and Figure 9 show that the divergence between $g_{S}$ and $g_{\mathrm{IQ}}$ is considerably reduced once a gain and phase synchronization procedure is performed. Due to this closeness, the proposed mitigation strategy can be applied to estimate the desired parameters. This has been verified in Figure 10, which shows the BER using the proposed estimation methodology.

### 3.3. Constellation Plots

**Figure 11:**
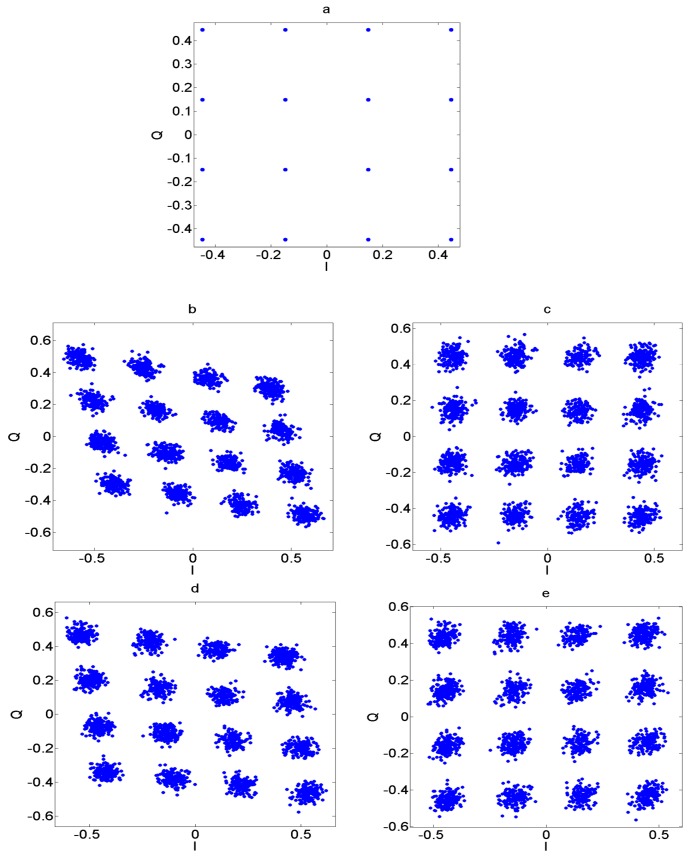
Constellation of 16 QAM signals under 20 dB SNR (**a**) transmitted signal (**b**) received signal and (**c**) corrected signal for *g* = *g*′ = 0.92 dB and *θ* = *θ*′ = −6 degrees; (**d**) Received and (**e**) corrected signal for *g* = 0.92 dB, *g*′ = 0.5 dB and *θ* = −6 degrees, *θ*′ = −2 degrees.

Figure 11 shows the constellations for the received and the corrected signal for a 16-QAM signal. The transceiver I/Q imbalance was applied to the signal in the presence of the AWGN channel. The degradation due to this gain and phase imbalance on the signal constellation can be seen in the figure. The proposed compensation technique is applied to the signal and the constellations are then observed. It can be seen that the proposed methodology improves the signal constellation and makes the symbol detection feasible at the receiver.

## 4. Discussion

Methods that deal with compensating the modulator impairments at the transmitter, using training signals require a feedback path to down-covert the RF signal to baseband or low IF. This feedback path contains a digital demodulator, an ADC and a frequency down-converter, which adds a time delay and phase mismatch to the received signal along with the ADC’s distortions. The output of the demodulator in the presence of these effects can be written as: $y_{\mathrm{delay}}=y\left(n-t_{d}\right)e^{j\phi_{d}}$, where $t_{d}$ is the time delay and $\phi_{d}$ is the phase shift caused by the feedback path. Prior to system identification, the input and output signals are time and phase aligned. An inaccurate adjustment leads to poor modeling and compensation performance by manifesting itself as dispersion in the gain and phase characteristics of the system.

For very small time delays, better time resolution (1/*f*_s_) is needed, which places a stringent requirement on the sampling frequency (*f*_s_) of the ADCs. In order to avoid changing the ADC or other hardware for increased sampling rate, signal processing techniques have been used in many works. A popular method for the time adjustment of these signals is the cross-correlation based method [30]. First, a coarse estimation is applied, followed by a Lagrange polynomial-based fine time delay estimation.

If we consider a function: $y_{l}=f\left(x_{l}\right)$, with values of the function known only at *l* = 1, 2, …, *P*, the Lagrange interpolation polynomial of degree *P*-1 can be used to find the values of *y* at other values of *x* [31]. Interpolation of the data using this polynomial is, however, a computationally complex procedure requiring O(*P*^2^) floating-point operations. Similarly, a phase shift adjustment needs to be performed before system identification. However, since the proposed methodology does not require the input/training signal for estimation of the imbalance parameters, it is not affected by the time delay and phase mismatch. Thus, cumbersome alignment techniques can be avoided using this method.

In addition to timing and phase mismatch, the feedback path can also add an *I*/*Q* imbalance to the received signal. Opting for a different receiver topology, instead of direct conversion, can solve this problem. For example, the method employed in [12] uses a super-heterodyne receiver in the feedback path to avoid receiver imbalance. However, this comes at a cost of hardware complexity and other feedback loop impairments, such as the gain/phase response of the feedback path; and, ADC distortions still need to be considered.

The method employed in [32] down-converts the RF signal to a low IF, followed by sampling and analog-to-digital conversion. Although, this can help avoid the impact of receiver imbalance and the proposed method can eliminate the effects of gain/phase and impulse response of the feedback loop, methods for timing, frequency and carrier synchronization are still required. The method proposed in [11] solves the problem of phase mismatch by using a diode detector in the feedback path of the transmitter, which calculates the instantaneous power. However, for the technique proposed in this work, no extra hardware in the transmitter is needed, as it does not need a feedback path at the transmitter, making it cost effective and very suitable especially for uplink scenarios. This advantage is due to the blind nature of the estimation procedure, eliminating the need for a feedback path and the resulting distortions.

## 5. Experimental Results

**Figure 12:**
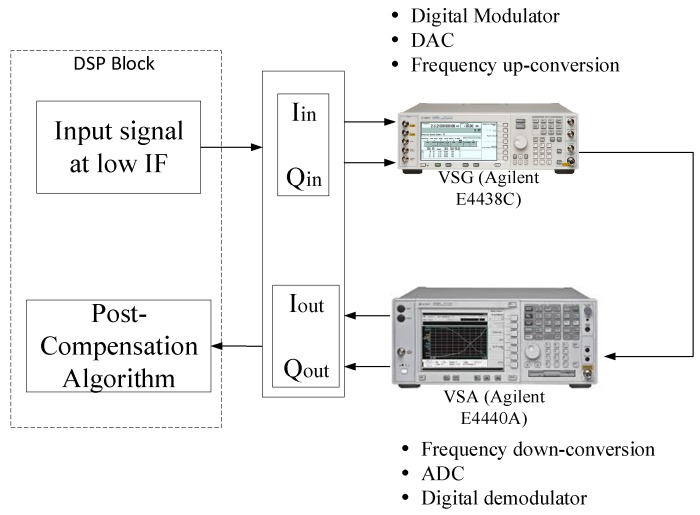
Measurement setup for evaluating the performance of proposed methodology.

After evaluating the performance of the proposed methodology using extensive simulations, the next step required experimental validation. The experimental setup is shown in Figure 12. Since the proposed mitigation strategy deals with frequency independent *I*/*Q* imbalances, the test signal selected was a 16-QAM signal with a narrow bandwidth of 10 KHz to minimize the effect of the frequency response, expected from the experimental setup. As many as 20,000 data points were generated and modulated using quadrature amplitude modulation. A raised cosine filter with a roll off factor of 0.3 and a filter delay of 3 was used to limit the bandwidth of the signal. The signal was then up-sampled by a factor of 8 (the sampling rate was 80 KHz). The *I* and *Q* components had the same variance and zero mean, thereby meeting the assumptions in Section 2.2. This signal was then shifted to a low IF of 20 KHz in the digital processing unit and loaded to a vector signal generator (VSG, Agilent E4438C, Keysight Technologies, Santa Rosa, CA, USA) through a general-purpose interface bus (GPIB). For direct conversion transceivers, the image lies exactly on the band of interest, and the image suppression cannot be seen clearly. Hence, conversion to low IF enables visualization of the image band.

**Figure 13:**
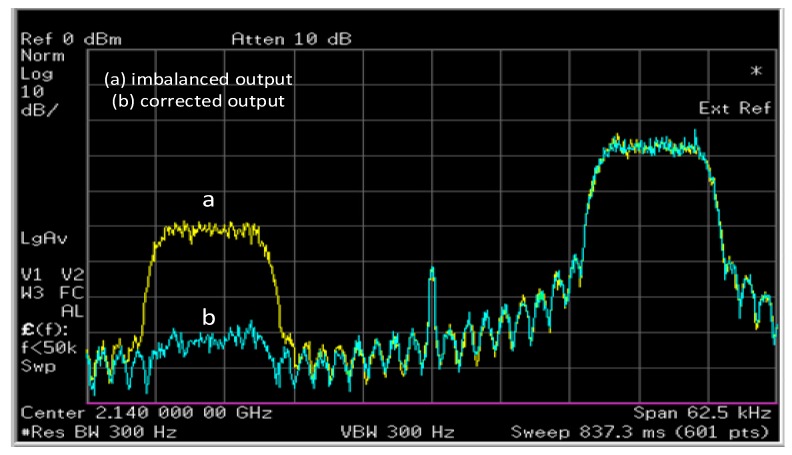
Measured output and post compensated signal.

**Figure 14:**
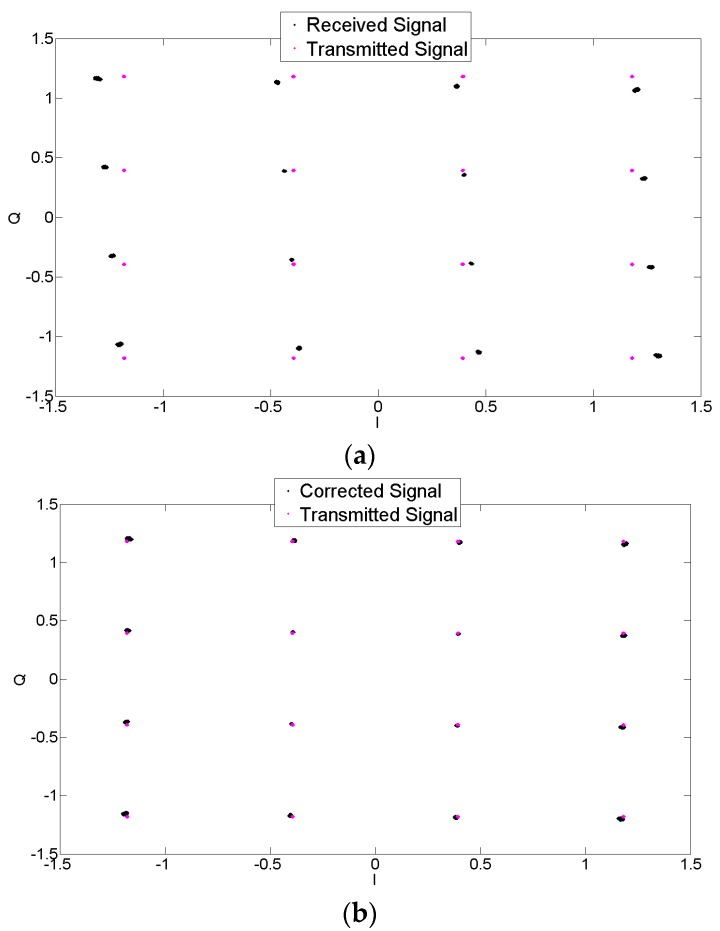
Constellation of 16 QAM (**a**) transmitted and received signals and (**b**) transmitted and corrected signals.

The VSG performed digital modulation, digital-to-analog conversion, and frequency up-conversion. In order to add modulator imperfections, a gain imbalance of 1 dB and a phase imbalance of 5 degrees were added to this signal to voluntarily add *I*/*Q* imbalance to the VSG. This signal was then transmitted at a carrier frequency of 2.14 GHz and captured using a vector signal analyzer (VSA, Agilent E4440A, Keysight Technologies, Santa Rosa, CA, USA) with a span of 62.5 KHz and a resolution bandwidth of 15.2 Hz. The VSA served as a receiver in this case. The receiver performed frequency down-conversion, analog-to-digital conversion and finally demodulation and adds its inherent imbalance. This captured signal, with a time length of 250 ms, was loaded into the digital signal processor without any compensation for time delay or phase mismatch. Finally, the proposed post compensation technique was applied to this signal. The imbalanced and corrected waveform were captured by the spectrum analyzer, and a snapshot of the spectrum is shown in Figure 13. It can be seen that before compensation, the image suppression was around −20 dB. However, after application of the proposed post-compensation algorithm, the image was reduced by around 30 dB, resulting in an image suppression of more than −50 dB. Finally, Figure 14 shows the corrupted and recovered constellation using the proposed methodology. It can be seen in Figure 13 that the inherent DC offset of the device still showed up in the compensated output. This issue can be resolved by subtracting the mean of the compensated signal from the signal, thus reducing the DC offset due to local oscillator leakage [32].

Further experiments were conducted using a 1 MHz signal with a sampling frequency of 5 MHz using the same values of gain and phase imbalance. Figure 14 shows the corrupted and recovered constellation using the proposed methodology for 1 MHz signal. It can be seen that the proposed methodology is able to mitigate the effects of *I*/*Q* imbalance and improve the signal constellation. This is also validated using the Error Vector Magnitude (EVM) [33] as shown in Table 4. It can be seen that the variation in EVM due to *I*/*Q* imbalance has been corrected using the proposed scheme and the NMSE is reduced considerably. The results obtained using the measurements were also compared to the simulations using the same values of imbalance parameters as in the measurement setup.

## 6. Conclusions

In this paper, the issue of transmitter and receiver *I*/*Q* imbalances has been addressed. Using statistical properties of the communication signals, an empirical PDF-based blind estimation method has been proposed to mitigate the effects of frequency independent *I*/*Q* imbalance in wireless transceivers.

A PDF of the impaired signal was estimated at the receiver, and the accuracy of this function was evaluated. Once the derived PDF was found to be accurate, the imbalance parameters were calculated by maximizing the PDF in terms of these parameters, resulting in four nonlinear equations. A nonlinear optimization technique was used to solve for the imbalance parameters, and the performance was evaluated.

Simulation results show that the proposed model resulted in reduced error, reasonable image suppression, and a very low BER, validating the modeling and estimation capability of the proposed method. The proposed model was also tested in a real-world experimental setup and performed reasonably well, meeting the desired mitigation performance.

## Figures and Tables

**Table 1:** Comparison of probability density functions (PDFs) for noiseless case and *g* = *g*′ = 0.92 dB and *θ* = *θ*′ = −6 degrees.

Signal	$D_{\mathrm{KL}}$		$D_{H}^{2}$	
	$f_{\mathrm{in},\mathrm{KDE}}||g_{\mathrm{KDE}}$	$f_{y}\left(y;Г\right)||g_{\mathrm{KDE}}$	$f_{\mathrm{in},\mathrm{KDE}}||g_{\mathrm{KDE}}$	$f_{y}\left(y;Г\right)||g_{\mathrm{KDE}}$
WCDMA 1111	1.146 × 10^−1^	2.2 × 10^−3^	2.38 × 10^−2^	4.75 × 10^−4^
LTE 101	1.368 × 10^−1^	2 × 10^−3^	2.8 × 10^−2^	4.97 × 10^−4^

**Table 2:** IMGsup using proposed Methodology for *g* = 0.92 dB, *g*′ = 0.5 dB and *θ* = −6 degrees, *θ*′ = −2 degrees under 20 dB SNR.

Signal	Image Suppression before Compensation (dB)	Image Suppression after Compensation (dB)
S1 (WCDMA 1101 with *f*_c_ around 10 MHz)	−16.2	−28.7
S2 (WCDMA 1101 with *f*_c_ around 5 MHz)	−15.5	−28.7
S3 (WCDMA 1101 with *f*_c_ around DC)	−15.4	−28.8

**Table 3:** Comparison of PDFs for *g* = *g*′ = 0.5 dB and *θ* = *θ*′ = −2 degrees under 20 dB SNR and Multipath Channel for 16-QAM OFDM signal.

	$D_{\mathrm{KL}}$	$D_{H}^{2}$
$g_{in}||g_{CH}$	0.0588	0.0135
$g_{in}||g_{IQ}$	0.0162	0.0042
$g_{IQ}||g_{S}$	0.0039	9.59 × 10^−4^

**Table 4:** NMSE and EVM for 1 MHz 16 QAM signal using experimental data and comparison with simulations.

EVM Uncompensated Signal	EVM (This Work)	EVM [15]	EVM (No imb.)	EVM (Sim.)	NMSE (dB) Uncompensated Signal	NMSE (This Work)	NMSE [15]	NMSE (No imb.)	NMSE (Sim.)
7.1651	0.7809	0.986	0.59	0.6162	−22.89	−41.77	−39.87	−43.96	−41.87

## Appendix A

To derive the closed form expression for the PDF of the output signal **y**, we start with the following PDF provided in Equation (16):

(A1) $$f_{y}(y;\Gamma )=\prod_{i=1}^{N}\frac{1}{2\pi \sqrt{\;|C_{y}(\Gamma )|}}\times \exp(-\frac{1}{2}y_{i}^{T}C_{y}^{-1}(\Gamma )y_{i})$$

The covariance matrix of **y** is given by:

(A2) $$C_{y}(\Gamma )=E(yy^{T})=E(Axx^{T}A^{T})=AC_{x}A^{T}$$

where $C_{x}$ is the covariance matrix of **x**. Replacing the expression of $C_{x}=\sigma^{2}I_{D}$ (using assumptions *A*1 and *A*2) in the above equation, we have

(A3) $$C_{y}(\Gamma )=\sigma_{}^{2}AA^{T}$$

The determinant of $C_{y}$ is givssssen by:

(A4) $$\left|C_{y}(\Gamma )|={\left(\sigma_{}^{2}\right)}^{R}|AA^{T}\right|$$

where *R* denotes the size of the matrix which is 2 in our case. Furthermore,

(A5) $$|C_{y}(\Gamma ){|}^{1/2}=\sigma_{}^{2}\gamma ;\gamma =\alpha_{1}\beta_{2}-\alpha_{2}\beta_{1}$$

The inverse of $C_{y}$ is given by:

(A6) $$C_{y}^{-1}(\Gamma )=\frac{1}{\sigma_{}^{2}}{\left(A^{T}\right)}^{-1}{\left(A\right)}^{-1}$$

where

(A7) $${\left(A^{T}\right)}^{-1}=\frac{1}{\gamma }\left(\begin{array}{cc} \beta_{2} & -\alpha_{2}\\ -\beta_{1} & \alpha_{1} \end{array}\right);A^{-1}=\frac{1}{\gamma }\left(\begin{array}{cc} \beta_{2} & -\beta_{1}\\ -\alpha_{2} & \alpha_{1} \end{array}\right)$$

Hence,

(A8) $$C_{y}^{-1}(\Gamma )=\frac{1}{\sigma_{}^{2}\gamma^{2}}\left(\begin{array}{cc} \beta_{2}{}^{2}+\alpha_{2}{}^{2} & -\beta_{1}\beta_{2}-\alpha_{1}\alpha_{2}\\ -\beta_{1}\beta_{2}-\alpha_{1}\alpha_{2} & \beta_{1}{}^{2}+\alpha_{1}{}^{2} \end{array}\right)$$

Putting the expressions for $C_{y}$^−1^ and |$C_{y}$|^1/2^ in Equation (A1), we obtain the closed form expression for the demodulated imbalanced signal as:

(A9) $$f_{y}(y;\Gamma )=\frac{1}{{\left(2\pi \sigma_{}^{2}|\gamma |\right)}^{N}}e^{-\left(\frac{1}{2\sigma_{}^{2}\gamma^{2}}\sum_{i=1}^{N}{\left(\beta_{2}I_{y}(i)-\beta_{1}Q_{y}(i)\right)}^{2}+{\left(\alpha_{1}Q_{y}(i)-\alpha_{2}I_{y}(i)\right)}^{2}\right)}$$

## Appendix B

In order to obtain the imbalanced parameters in **A** in Equation (11), we derivate the *log*_e_ of the obtained PDF of the imbalanced signal and equate it to zero i.e., $\frac{\partial \ln\left(f\left(I_{y},Q_{y}\right)\right)}{\partial \alpha_{1}}=0$, providing the following expression:

(A10) $$\sum_{i=1}^{N}\beta_{2}p(i)-\sum_{i=1}^{N}\gamma Q_{y}(i)p_{2}(i)=N\beta_{2}\sigma^{2}\gamma^{2}$$

Similarly $\frac{\partial \ln\left(f\left(I_{y},Q_{y}\right)\right)}{\partial \sigma }=0$ results in:

(A11) $$\sum_{i=1}^{N}p(i)=2N\sigma^{2}\gamma^{2}$$

Placing Equation (A11) into Equation (A10), we get:

(A12) $$\frac{\beta_{2}}{2}\sum_{i=1}^{N}p(i)-\gamma \sum_{i=1}^{N}p_{2}(i)Q_{y}(i)=0$$

Similarly, derivating the *log_e_* of the PDF with respect to the remaining imbalance parameters leads to the following equations:

(A13) $$\frac{\alpha_{2}}{2}\sum_{i=1}^{N}p(i)-\gamma \sum_{i=1}^{N}p_{1}(i)Q_{y}(i)=0$$

(A14) $$\frac{\alpha_{1}}{2}\sum_{i=1}^{N}p(i)-\gamma \sum_{i=1}^{N}p_{1}(i)I_{y}(i)=0$$

(A15) $$\frac{\beta_{1}}{2}\sum_{i=1}^{N}p(i)-\gamma \sum_{i=1}^{N}p_{2}(i)I_{y}(i)=0$$

These equations in vector format have been presented in Equations (24)–(27). The definitions for *p*(*i*), *p*_1_(*i*) and *p*_2_(*i*) have been provided in Equations (18)–(20).

## Appendix C

Here we provide the Cramer-Rao lower bound (CRLB) for the proposed estimator using the Fisher information matrix. The variance of the proposed estimator is given by:

(A16) $$var(\hat{\Gamma })\geq {\left[I(\Gamma )\right]}_{mn}{}^{-1}$$

where ${\left[I(\Gamma )\right]}_{mn}$ is the Fisher information matrix. From Equation (A9), the log of the likelihood function can be obtained as:

(A17) $$ln(f_{y}(y;\Gamma ))=-Nln(2\pi \sigma^{2})-Nln(\gamma )-\frac{1}{2\sigma^{2}}\sum_{i}y_{i}^{T}{\left(AA^{T}\right)}^{-1}y_{i}^{}$$

The Hessian of this log likelihood function is given by:

(A18) $${\left[H(\Gamma )\right]}_{mn}=\frac{\partial^{2}ln(f_{y}(y;\Gamma ))}{\partial \Gamma_{m}\partial \Gamma_{n}}={\left[H_{1}\right]}_{mn}+{\left[H_{2}\right]}_{mn}$$

where **H**_1_ and **H**_2_ are the Hessian matrices of the second and third term, respectively. Also:

(A19) $${\left[I(\Gamma )\right]}_{mn}=-E({\left[H(\Gamma )\right]}_{mn})$$

(A20) $$E({\left[H_{1}\right]}_{mn})=E(\frac{N}{\gamma^{2}}\frac{\partial \gamma }{\partial \Gamma }{\frac{\partial \gamma }{\partial \Gamma }}^{T})=\frac{N}{\gamma^{2}}\left(\begin{array}{cccc} \beta_{2}{}^{2} & -\alpha_{2}\beta_{2} & -\beta_{1}\beta_{2} & \alpha_{1}\beta_{2}\\ -\alpha_{2}\beta_{2} & \alpha_{2}^{2} & \alpha_{2}\beta_{1} & -\alpha_{1}\alpha_{2}\\ -\beta_{1}\beta_{2} & \alpha_{2}\beta_{1} & \beta_{1}{}^{2} & -\alpha_{1}\beta_{1}\\ \alpha_{1}\beta_{2} & -\alpha_{1}\alpha_{2} & -\alpha_{1}\beta_{1} & \alpha_{1}^{2} \end{array}\right)$$

Using the following statistics of the received signal:

(A21) $$E[Q_{y}^{2}]=(\alpha_{2}^{2}+\beta_{2}^{2})\sigma^{2}$$

(A22) $$E[I_{y}^{2}]=(\alpha_{1}^{2}+\beta_{1}^{2})\sigma^{2}$$

(A23) $$E[I_{y}Q_{y}]=(\alpha_{1}\alpha_{2}^{}+\beta_{1}\beta_{2}^{})\sigma^{2}$$

The expected value of the second Hessian matrix can be written as:

(A24) $$E({\left[H_{2}\right]}_{mn})=-\frac{N}{\gamma^{2}}\left(\begin{array}{cccc} \alpha_{2}^{2}+\beta_{2}^{2} & 0 & -(\alpha_{1}\alpha_{2}+\beta_{1}\beta_{2}) & 0\\ 0 & \alpha_{2}^{2}+\beta_{2}^{2} & 0 & -(\alpha_{1}\alpha_{2}+\beta_{1}\beta_{2})\\ -(\alpha_{1}\alpha_{2}+\beta_{1}\beta_{2}) & 0 & \alpha_{1}^{2}+\beta_{1}^{2} & 0\\ 0 & -(\alpha_{1}\alpha_{2}+\beta_{1}\beta_{2}) & 0 & \alpha_{1}^{2}+\beta_{1}^{2} \end{array}\right)$$

(A25) $${\left[I(\Gamma )\right]}_{mn}=\frac{N}{\gamma^{2}}\left(\begin{array}{cccc} \alpha_{2}^{2} & \alpha_{2}\beta_{2} & -\alpha_{1}\alpha_{2} & -\alpha_{1}\beta_{2}\\ \alpha_{2}\beta_{2} & \beta_{2}^{2} & -\alpha_{2}\beta_{1} & -\beta_{1}\beta_{2}\\ -\alpha_{1}\alpha_{2} & -\alpha_{2}\beta_{1} & \alpha_{1}^{2} & \alpha_{1}\beta_{1}\\ -\alpha_{1}\beta_{2} & -\beta_{1}\beta_{2} & \alpha_{1}\beta_{1} & \beta_{1}^{2} \end{array}\right)=\frac{N}{\gamma^{2}}\Lambda$$

Having gained the expression for the Fisher information matrix using Equation (A25), the variance of the proposed estimator can be calculated by Equation (A16) as:

(A26) $$var(\hat{\Gamma })\geq \frac{\gamma^{2}}{N\Lambda }$$
